# Supplementary material for: A Global Screen for Assembly State Changes of the Mitotic Proteome by SEC-SWATH-MS
Source: Cell Syst. 2020 Feb 26;10(2):133–155.e6. doi: 10.1016/j.cels.2020.01.001 (PMC7042714; doi:10.1016/j.cels.2020.01.001)

Q7L2H7 | EIF3M_HUMAN | EIF3M HFLB5 PCID1 GA17 PNAS−125
Monomer MW [kDa]: 42.503 Monomer expected elution fraction: 48

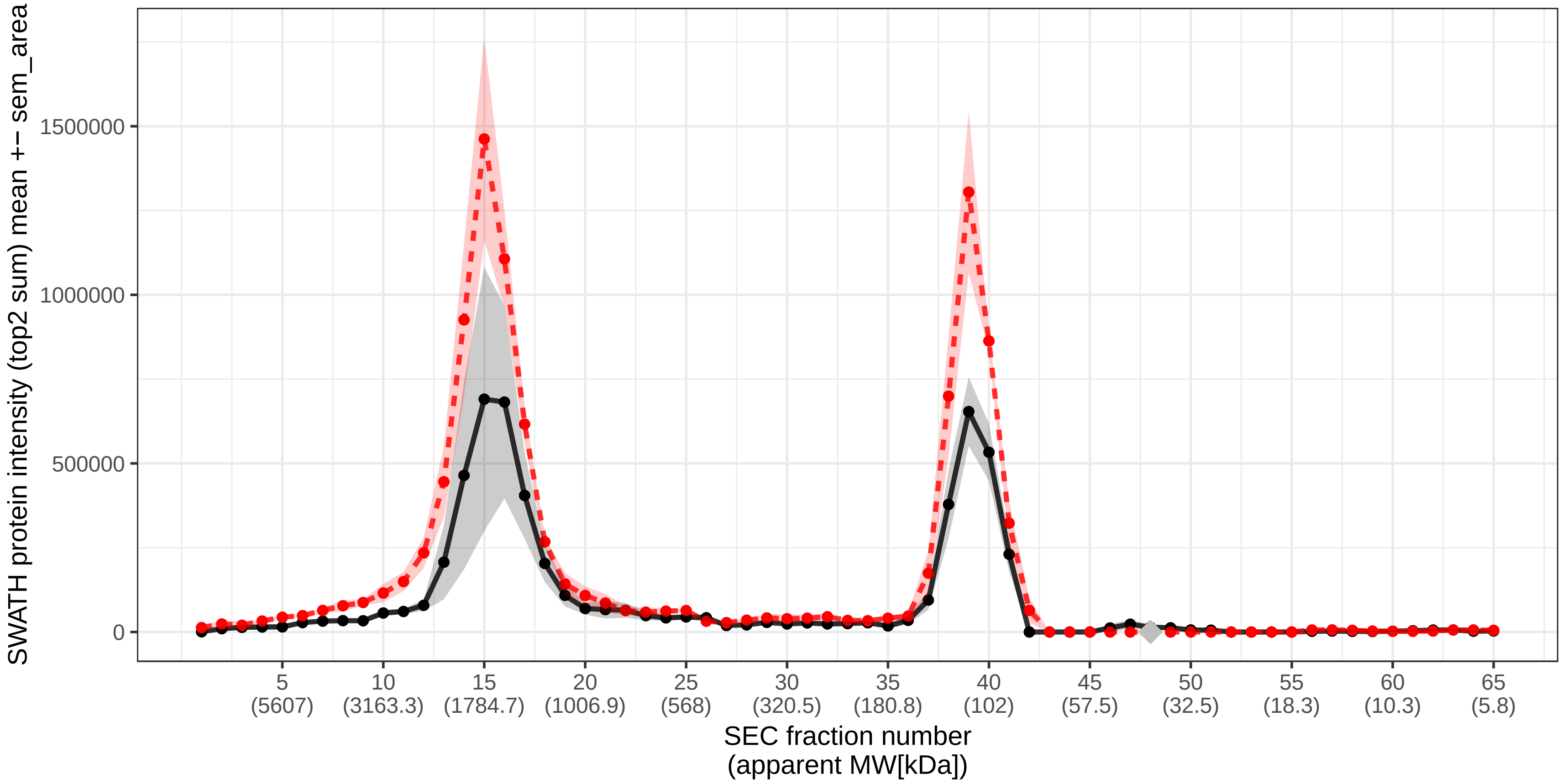

Supplement: Data S1. SEC-SWATH-MS Protein Chromatograms, Related to Figure 1 [file mmc6.zip › SECchrom_Q7L2H7_EIF3M_HUMAN_EIF3M_HFLB5_PCID1_GA17_PNAS-125.pdf]
